# Supplementary material for: Complex introgression among three diverged largemouth bass lineages
Source: Evol Appl. 2021 Nov 9;14(12):2815–30. doi: 10.1111/eva.13314 (PMC8674896; doi:10.1111/eva.13314)
Supplement: Supplementary file 1 — Supplementary Material [file EVA-14-2815-s002.docx]

## Supplementary File 1

**Supp. Table 1: Sample information for GBS individuals.** Population genetic summary statistics are provided for GBS sampling sites with at least 5 individuals using 8,582 SNPs. H_o_: observed heterozygosity averaged across loci; H_e_: expected heterozygosity averaged across loci; F_IS_: Wright's F‐statistics averaged across loci (Nei & Chesser, 1983)

| Population | Group | H_o_ | H_e_ | F_IS_ | N | Latitude | Longitude |
| --- | --- | --- | --- | --- | --- | --- | --- |
| Lake Allatoona, GA | ILB | 0.277 | 0.295 | 0.062 (0.054-0.072) | 9 | 34.1331 | -84.6244 |
| Lake Guntersville, AL | ILB | 0.346 | 0.324 | -0.068  (-0.075--0.060) | 10 | 34.5523 | -86.1192 |
| Lay Lake, AL | ILB | 0.252 | 0.286 | 0.118  (0.110-0.127) | 9 | 33.1166 | -86.482 |
| Lake Eufaula, AL | ILB | 0.276 | 0.291 | 0.051  (0.040-0.062) | 5 | 31.91168 | -85.15915 |
| Lake Harding, AL | ILB | 0.315 | 0.310 | -0.016  (-0.028 -0.004) | 5 | 32.6911 | -85.1114 |
| Rocky Mountain PFA, GA | ILB | 0.287 | 0.305 | 0.059  (0.0475-0.072) | 5 | 34.34236 | -85.32919 |
| Big Bayou Canot, AL | DLB | 0.145 | 0.150 | 0.032  (0.015-0.048) | 6 | 30.83575 | -87.98424 |
| D’Olive Bay, AL | DLB | 0.140 | 0.145 | 0.036  (0.021-0.050) | 8 | 30.65620 | -87.41795 |
| Sipsey River, AL | DLB | 0.167 | 0.171 | 0.020  (0.011-0.031) | 13 | 33.91454 | -87.68724 |
| St. Johns River, FL | FLB | 0.122 | 0.122 | -0.0004  (-0.015- 0.013) | 17 | 28.24763 | -81.38371 |
| Hatchery FLB | FLB | 0.093 | 0.105 | 0.103  (0.079-0.127) | 7 | NA | NA |
| FL Bass Conservation Center | FLB | 0.114 | 0.112 | -0.015  (-0.038 -0.007) | 5 | 28.50138 | -82.05196 |
| Lake Mattoon, IL | NLB | 0.139 | 0.136 | -0.02  (-0.032 - -0.007) | 10 | 39.34934 | -88.47698 |
| Hatchery NLB | NLB | 0.125 | 0.128 | 0.022  (0.006-0.039) | 6 | NA | NA |
| Otter Lake, IL | NLB | 0.139 | 0.143 | 0.031  (0.018-0.044) | 10 | 39.41576 | -89.90370 |
| Sugar Lake, MN | NLB | 0.077 | 0.080 | 0.031  (0.011-0.050) | 9 | 45.31384 | -94.04487 |
| Lamar County Lake, AL | ILB | NA | NA | NA | 1 | 33.7769 | -88.2349 |
| Reeves Branch, Chattahoochee R., AL | ILB | NA | NA | NA | 5 | 31.88108 | -85.1875 |
| Reelfoot Reservoir, TN | ILB | NA | NA | NA | 4 | 36.40582 | -89.3809 |
| Tensaw Lake, AL | DLB | NA | NA | NA | 2 | 31.03156 | -87.90164 |

**Supp. Table 2.** Accuracy and efficiency of 73 SNP panel when simulated 4th generation backcrossed individuals are included. Number of simulated individuals (rows), which were assigned to one of three lineages or into a hybrid category (columns). Simulated individuals were assigned based on their STRUCTURE Q-values, with a threshold of Q ≥ 0.94 for pure individuals. Parameters of efficiency, accuracy and overall performance of the assignment method are given in percent. Individuals correctly assigned are in bold type. DLB: Delta Largemouth Bass (*M. salmoides*), NLB: northern Largemouth Bass (*M. salmoides*), FLB: Florida Bass (*M. floridanus*); BX: backcrosses.

| Simulated/ Assigned | DLB | FLB | NLB | DLB-FLB | DLB-NLB | NLB-FLB | Triple Hybrid | Total Simulated |
| --- | --- | --- | --- | --- | --- | --- | --- | --- |
| NB-FL BX4 |  | 27 | 26 |  | 3 | **43** | 1 | 100 |
| DB-NB BX4 | 19 | 18 |  |  | **63** |  |  | 100 |
| DB-FL BX4 | 28 | 27 |  | **45** |  |  |  | 100 |
| Total | 47 | 72 | 26 | 45 | 66 | 43 | 1 |  |
| Efficiency | 100% | 100% | 100% | 88.40% | 92.40% | 82.80% | 98.67% |  |
| Accuracy | 67.11% | 58.14% | 78.74% | 98.88% | 98.93% | 100.00% | 91.36% |  |
| Performance | 67.11% | 58.14% | 78.74% | 87.41% | 91.41% | 82.80% | 90.14% |  |


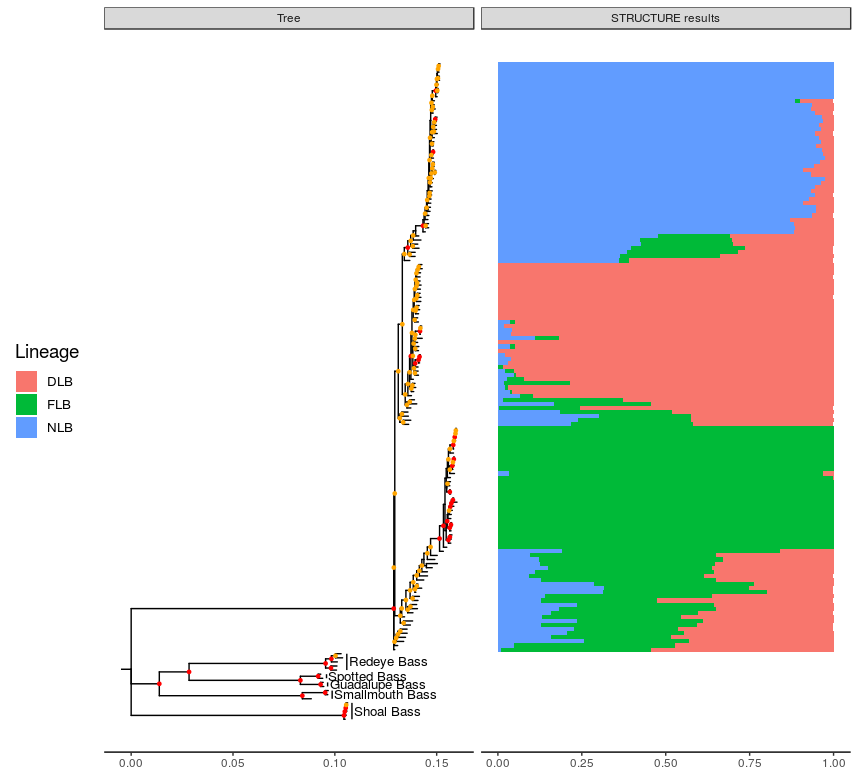


**Supp. Fig. 1.** Maximum likelihood phylogeny constructed using 18,538 concatenated GBS SNPs and IQ-TREE (1000 ultrafast bootstrap replicates). Nodes in red indicate 100% ultrafast bootstrap support, nodes in yellow indicate 50-99% ultrafast bootstrap support. STRUCTURE plots (K=3) are included and show the ancestry membership proportions (Q-values) for the three largemouth bass lineages as inferred using 8,582 SNPs (DLB=Delta Largemouth Bass, NLB=northern Largemouth Bass, FLB=Florida Bass).


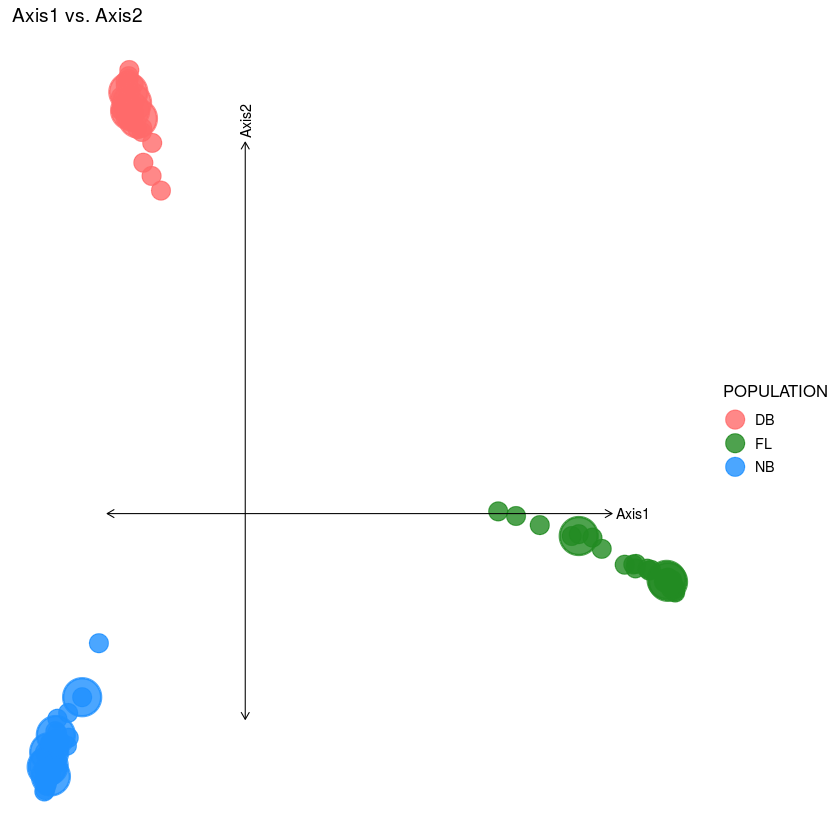


**Supp. Figure 2:** Principal component analysis on 100 individuals based on 2,180 diagnostic SNPs. Individuals are colored by lineage (DB: Delta Largemouth Bass, FL: Florida Bass, NB: northern Largemouth Bass). The top 500 SNPs based on PC loadings were used to design the 73 SNP diagnostic panel. PC1: 72.7% of variation, PC2: 13.3% of variation.


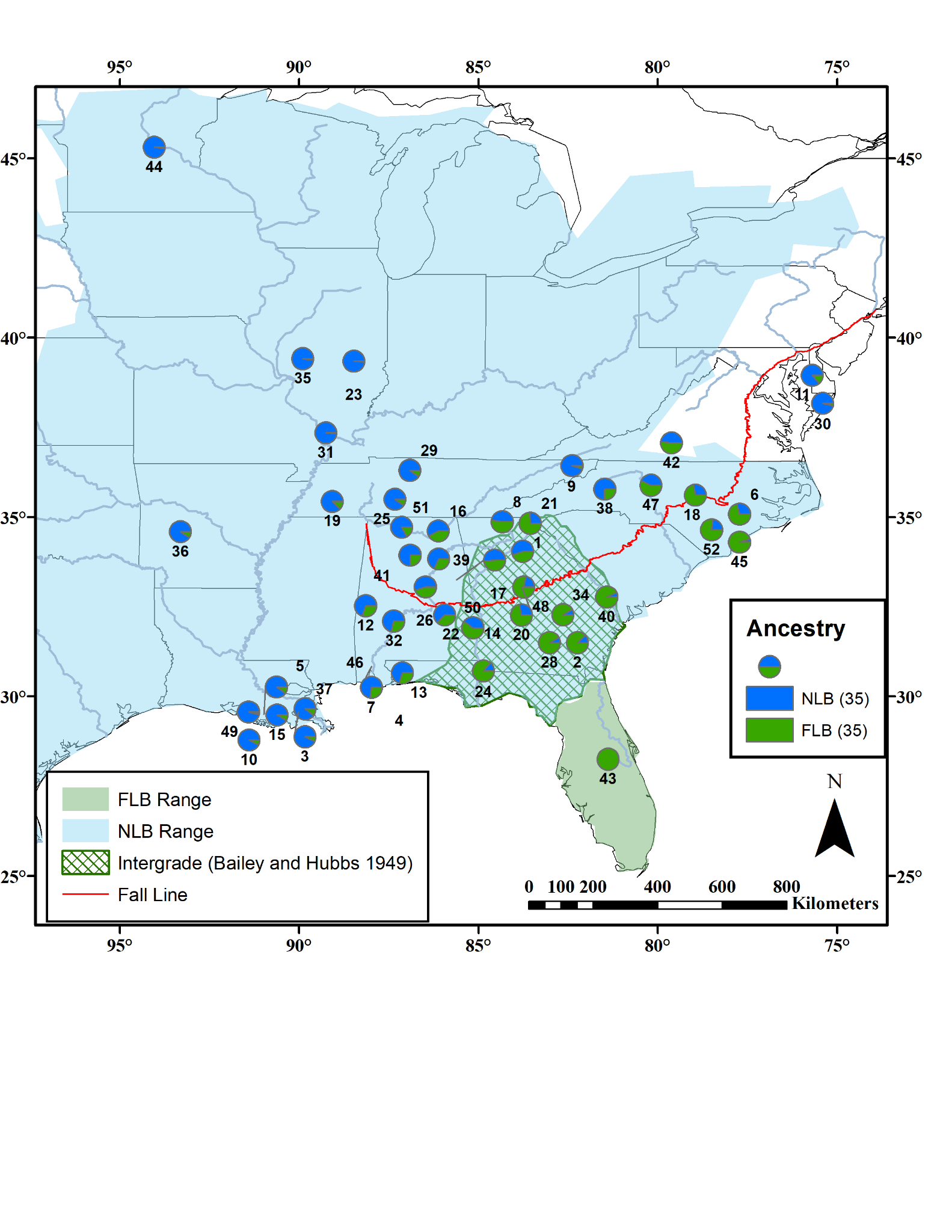


**Supp. Figure 3.** Sampling sites of largemouth bass assayed with 35 SNP panel diagnostic for northern Largemouth Bass (NLB) and Florida Bass (FLB). Populations are labelled as in Supp. File 2 and represented by pie graphs showing the mean estimated ancestry proportions for the two bass lineages based on counting the number of FLB and NLB alleles. Accepted native ranges of NLB and FLB are shown, as well as the NLB-FLB hybrid zone as described by Bailey & Hubbs, (1949). Map data from ESRI; FLB and NLB native ranges from Taylor et al., (2019).

**Supp. File 2.** Sampling sites assayed with 73 SNP panel and 35 SNP FLB–NLB panel. Numbers correspond to labels in Figure 1 and Supp. Figure 3, ancestry proportions are based on STRUCTURE results (K=3), using reference individuals.

**Supp. File 3.** Primer sequences (forward, reverse, and extension) designed to genotype two panels (52 and 39 SNPs using iPLEX PCR on the Agena MassARRAY System. 18 SNPs failed QC and are noted, however all primers must be included when genotyping with the MassARRAY platform and filtered after.
